# Increased Gray Matter Volume and Resting-State Functional Connectivity in Somatosensory Cortex and their Relationship with Autistic Symptoms in Young Boys with Autism Spectrum Disorder

**DOI:** 10.3389/fphys.2017.00588

**Published:** 2017-08-15

**Authors:** Jia Wang, Kuang Fu, Lei Chen, Xujun Duan, Xiaonan Guo, Heng Chen, Qiong Wu, Wei Xia, Lijie Wu, Huafu Chen

**Affiliations:** ^1^Department of Children's and Adolescent Health, Public Health College of Harbin Medical University Harbin, China; ^2^Department of MR Diagnosis, The Second Affiliated Hospital of Harbin Medical University Harbin, China; ^3^Key Laboratory for NeuroInformation of Ministry of Education, School of Life Science and Technology and Center for Information in BioMedicine, University of Electronic Science and Technology of China Chengdu, China

**Keywords:** autism spectrum disorder (ASD), gray matter volume (GMV), voxel-based morphometry (VBM), functional connectivity, resting-state

## Abstract

Autism spectrum disorder (ASD) has been widely recognized as a complex neurodevelopmental disorder. A large number of neuroimaging studies suggest abnormalities in brain structure and function of patients with ASD, but there is still no consistent conclusion. We sought to investigate both of the structural and functional brain changes in 3–7-year-old children with ASD compared with typically developing controls (TDs), and to assess whether these alterations are associated with autistic behavioral symptoms. Firstly, we applied an optimized method of voxel-based morphometry (VBM) analysis on structural magnetic resonance imaging (sMRI) data to assess the differences of gray matter volume (GMV) between 31 autistic boys aged 3–7 and 31 age- and handness-matched male TDs. Secondly, we used clusters with between-group differences as seed regions to generate intrinsic functional connectivity maps based on resting-state functional connectivity magnetic resonance imaging (rs-fcMRI) in order to evaluate the functional impairments induced by structural alterations. Brain-behavior correlations were assessed among GMV, functional connectivity and symptom severity in children with ASD. VBM analyses revealed increased GMV in left superior temporal gyrus (STG) and left postcentral gyrus (PCG) in ASD children, comparing with TDs. Using left PCG as a seed region, ASD children displayed significantly higher positive connectivity with right angular gyrus (AG) and greater negative connectivity with right superior parietal gyrus (SPG) and right superior occipital gyrus (SOG), which were associated with the severity of symptoms in social interaction, communication and self-care ability. We suggest that stronger functional connectivity between left PCG and right AG, SPG, and SOG detected in young boys with ASD may serve as important indicators of disease severity. Our study provided preliminary functional evidence that may underlie impaired higher-order multisensory integration in ASD children.

## Introduction

Autism spectrum disorder (ASD) is characterized by deficits in social communication, social interaction, and restricted, repetitive patterns of behavior, interests, or activities as defined in the fifth edition of the Diagnostic and Statistical Manual of Mental Disorders (DSM-5) (American Psychiatric Association, 2013). The prevalence of ASD seems to be increasing with time around the world. According to the Centers for Disease Control and Prevention (CDC) report, ASD affected nearly 1 in 68 children in the United States in 2012 (CDC, 2016). This estimate is significantly higher than the estimate of 1 in 152 children in 2002 (CDC, 2007). Information from China has not figured prominently in the prevalence estimates for ASD, but two previous reviews based on published provincial epidemiological studies also reported that the prevalence of ASD has increased from 10.3 per 10,000 in 2009 (Sun and Allison, 2009) to a high of 26.6 per 10,000 in 2013 (Sun et al., 2013) in mainland China. There is no doubt that the number of ASD patients is growing rapidly, which is accompanied by a huge burden on the caregivers as well as the whole society.

Autism spectrum disorder (ASD) has been widely recognized as a complex neurodevelopmental disorder. The most coherent finding from Magnetic Resonance Image (MRI) studies suggest a atypical neurodevelopmental trajectory of global volumes in ASD, characterized by an early brain overgrowth in childhood, followed by arrested growth in early adolescence, and possibly a decrease later in adulthood (Courchesne et al., 2011; Ha et al., 2015; Lange et al., 2015; Lin et al., 2015). The hypothesis of early brain overgrowth in children with ASD around 2–4 years of age is currently one of the most prominent theories on the neuroimaging of ASD, which is confirmed by evidences from both case-control (Courchesne et al., 2001; Sparks et al., 2002) and longitudinal MRI studies (Schumann et al., 2010). Since the cerebral cortex starts changing in the autistic brains early in life, it is of critical importance to examine how the brain develops over this growth epoch.

Apart from the obvious age effects of whole brain volume, another general finding of structural imaging studies is that volumetric alterations of gray matter in ASD distributed widely in brain and also differed remarkably by age. The latest meta-analysis (Liu et al., 2017) aimed to identify the specific gray matter abnormalities in pediatric ASD individuals suggested that pediatric ASD individuals showed significant gray matter increases in the right angular gyrus (AG), left superior and middle frontal gyrus, left precuneus, left inferior occipital gyrus and right inferior temporal gyrus and decreases in the left cerebellum and left postcentral gyrus (PCG). While another meta-analysis (Duerden et al., 2012) suggested that children/adolescents were more likely than adults to have increased gray matter in bilateral fusiform gyrus, right cingulate and insula, which is greatly inconsistent with the former. It is difficult to draw a definitive conclusion from previous studies. The neuroanatomical abnormities existed in the ASD children are still an issue needs to be explored.

Atypical brain anatomy and neurodevelopment will inevitably leads to functional changes. Over the past few decades, the claim that ASD is characterized by disrupted functional connectivity has been reliably supported by evidence from resting-state functional connectivity magnetic resonance imaging (rs-fcMRI) studies, even though with mixed findings (Rane et al., 2015). Some studies suggests that patients with ASD showed anatomical distance-dependent alterations in functional connectivity between different brain regions (Long et al., 2016), indicated an increased connectivity within local and short-distance brain regions (Supekar et al., 2013; Itahashi et al., 2015), with respect to reduced connectivity between long-range brain regions in particular between frontal and posterior-temporal cortical systems which play key roles in social-affective information processing (Schmitz et al., 2008). Other studies have found that participants at different development stages could surely make a difference in the functional connectivity of ASD. Hyper-connectivity may be more characteristic of young children with ASD, while hypo-connectivity may begin to emerge in adolescence and persist into adulthood (Uddin et al., 2013; Nomi and Uddin, 2015). Despite the early developmental origins of this disorder and its variable developmental trajectory, almost all of the current literature on brain functional connectivity has focused on adolescents and adults with ASD, rather than children. To our knowledge, only few rs-fcMRI studies have examined young children with ASD and most of them concerned the functional connectivity between brain regions important for social communication and language (Dichter, 2012; Shen et al., 2016), while the neural mechanisms underlying peculiar behaviors of ASD has not received enough attention. The degree to which the altered connectivities are predictive and specific for the abnormal behaviors is unknown.

Although abnormalities of the gray matter volume (GMV) and functional connectivity in ASD differ at different development stages, it's now abundantly clear that these alterations are most prominent during childhood. Therefore, we focused on a narrow age range of 3–7 year-old and limited the gender for male in order to minimize developmental differences between different age groups and genders (Carper et al., 2002; Bloss and Courchesne, 2007). Our study specifically aimed to reveals the abnormal structural and functional pattern in ASD children. We used a combined methods including voxel-based morphometric (VBM) technique along with rs-fcMRI to investigate whether brain regions with structural alterations in ASD children also existing abnormal functional connectivity. Our findings will contribute to the understanding of the neural basis of some abnormal behaviors in ASD children and will provide imaging evidence for early diagnosis and establishment of a specific intervention program. We hypothesized that there will be abnormal GMV and functional connectivity in several brain regions in children with ASD, in comparison with matched typically developing controls (TDs), and that these alterations in structure and connectivity would be associated with increased symptom severity.

## Materials and methods

### Participants

Thirty one male ASD children and 31 age-, handness-matched male TDs were recruited in this study. All children underwent a high-resolution T1 structure MRI scan. 25 children with ASD and 27 TDs underwent a resting-state fMRI scan (Table 1). All subjects with ASD were recruited from the Children Development and Behavior Research Center of Harbin Medical University. TDs were recruited through local kindergartens. None of them were on psychotropic medications or had a reported history of any severe medical problem or any neurological or psychiatric condition. Written informed consents were obtained from every participant's guardian after fully explaining the purpose of this study. This study was approved by the ethics review committee of Harbin Medical University.

**Table 1:** Demographic data (means ± SD) of ASD and typically developing controls.

**Subject characteristics**	**VBM**			**rs-fcMRI**		
	**ASD**	**TD**	***p*-value**	**ASD**	**TD**	***p*-value**
*n*	31	31		25	27	
Age, years	4.83 ± 1.12	4.83 ± 0.85	0.996	4.83 ± 1.20	4.74 ± 0.87	0.775
Age rang, years	3.47–7.93	3.18–6.18		3.47–7.93	3.18–6.18	
Male/female	31/0	31/0		25/0	27/0	
Right/left-handed	24/7	28/3	0.167	19/6	24/3	0.389
PPVT	62.52 ± 17.64	97.13 ± 27.71	0.000^*^	59.88 ± 16.03	98.11 ± 27.91	0.000^*^
ABC total score	59.83 ± 31.43	N/A	N/A	64.46 ± 32.10	N/A	N/A
**ABC SUB-SCALE**						
Sensory	7.87 ± 4.91	N/A	N/A	8.54 ± 4.75	N/A	N/A
Relating	14.27 ± 7.83	N/A	N/A	15.17 ± 7.91	N/A	N/A
Stereotypes and object use	9.60 ± 7.86	N/A	N/A	10.83 ± 8.05	N/A	N/A
Language	15.37 ± 8.83	N/A	N/A	16.42 ± 9.35	N/A	N/A
Self-help and social	12.73 ± 7.41	N/A	N/A	13.50 ± 7.63	N/A	N/A
**ADOS SUB-SCALE**						
Communication	5.90 ± 1.81	N/A	N/A	6.08 ± 1.82	N/A	N/A
Social interaction	9.23 ± 2.40	N/A	N/A	9.54 ± 2.57	N/A	N/A
Communication+social interaction	15.13 ± 3.74	N/A	N/A	15.62 ± 3.89	N/A	N/A
Stereotyped behaviors and restricted interests	1.90 ± 1.21	N/A	N/A	2.04 ± 1.12	N/A	N/A
**ADI-R SUB-SCALE**						
Social Interaction	22.21 ± 3.44	N/A	N/A	22.03 ± 3.55	N/A	N/A
Communication	14.92 ± 4.20	N/A	N/A	15.13 ± 4.18	N/A	N/A
Restricted, Repetitive, and Stereotyped Behaviors	7.54 ± 2.28	N/A	N/A	7.07 ± 2.55	N/A	N/A

*ASD, Autism spectrum disorder; TD, typically developing; PPVT, Peabody Picture Vocabulary Test; ABC, Autism Behavior Checklist; ADOS, Autism Diagnostic Observation Schedule; ADI-R, Autism Diagnostic Interview-Revised; N/A, not applicable (TDs did not receive the ABC, ADOS, and ADI-R); SD, standard deviation; ABC, ADOS, and ADI-R scores were not available for one ASD participant, therefore n, 30 for VBM analysis and n, 24 for resting-state functional connectivity analysis in ASD group*.

* *Indicates statistical significance p < 0.001*.

### Diagnosis and clinical assessment

Diagnosis of autistic disorder were based on the fifth edition of the Diagnostic and Statistical Manual of Mental Disorders (DSM-5) combined with a series of clinical assessments including a detailed developmental history, clinical observation and cognitive condition and were supported by the Autism Diagnostic Interview-Revised (ADI-R) (Lord et al., 1994) and the Autism Diagnostic Observation Schedule (ADOS) (Lord et al., 2000). Exclusion criteria included known psychiatric, neurological (e.g., epilepsy, Tourette's syndrome), or genetic disorders (eg, fragile X, Rett syndrome), a history of a loss of consciousness for more than 5 min and those currently taking psychoactive medication.

In addition, we chose Peabody Picture Vocabulary Test (PPVT) (Dunn et al., 2006) in order to estimate the overall intelligence level in the children of our sample, given that it does not require an oral or written response, and can be rapidly administered and utilized to evaluate people with language problems. Parents were asked to fill out the Autism Behavior Checklist (ABC) (Krug et al., 1980), a scale consists 57 items about the atypical behaviors of ASD patients and these behaviors are related to five domains (sensory, relating, stereotypes and object use, language, and self-help and social).

### Data acquisition

For each participant, we acquired MRI data at the Department of MR Diagnosis of the Second Hospital affiliated of Harbin Medical University, using a 3.0 Tesla Achieva Magnetic Resonance System (Philips, The Netherlands). All MRI scans were performed under sedation using chloral hydrate, followed a strict clinical protocol. A caregiver for each participant was present throughout the duration of the scan.

Anatomical T1 images of the whole brain were scanned with a volumetric three-dimensional spoiled gradient recall sequence: repetition time (*TR*) = 8.5 ms; echo time *(TE)* = 3.9 ms; flip angle = 9°; voxel size = 1 × 1 × 1 mm; 176 axial slices. Resting-state fMRI images were acquired via a gradient-echo echo-planar pulse sequence: *TR* = 2,000 ms; *TE* = 30 ms; flip angle = 90°; 39 axial slices; slice thickness = 3 mm (with 1 mm gap); field of view (FOV) = 240 × 240 mm; voxel size = 3.75 × 3.75 × 4 mm. 210 volumes resulting in 7 min of data were obtained.

### Data analysis

#### Voxel-based morphometry analysis

A VBM analysis was applied on the T1 images to obtain the GMV of each brain regions (Ashburner and Friston, 2000). It was performed using the Computational Anatomy Toolbox (CAT12 http://dbm.neuro.uni-jena.de/cat/) which is based on the Statistic Parameter Map package (SPM12 http://www.fil.ion.ucl.ac.uk/spm). First we manually coregistered all T1 images to set the anterior commissure at the origin of the Montreal Neurological Institute (MNI) coordinate system. The coregistered images were segmented into gray matter (GM), white matter (WM), and cerebrospinal fluid (CSF). As our images were obtained from young children, the tissue probability maps of GM, WM, and CSF of children in this age range were obtained using the Template-O-Matic Toolbox (TOM8 http://dbm.neuro.uni-jena.de/software/tom/). A diffeomorphic non-linear registration algorithm (DARTEL) was used to create a customized T1 template of our own images rather than a standard T1 template and the segmented GM and WM maps were spatially normalized. The resulting images were then normalized to MNI space using an affine spatial normalization. A further modulation was applied to convert the voxel values of tissue concentration (density) to measures of volume. Finally the normalized GM maps were smoothed with an isotropic Gaussian kernel (full width at half maximum = 8 mm).

A voxel-by-voxel two sample *t*-test was applied to compare the GMV between ASD group and TD group based on the general linear model using the SPM12 toolbox. Then an Alphasim multiple correction was applied on the resulted T map with voxel level *p* < 0.001 and cluster level *p* < 0.05.

The GMV of the clusters which showed significant difference between ASD and TD were extracted as the mean GMV values of all voxels in the corresponding cluster. Then a Pearson correlation analysis was used to determine the relationship between GMV of the atypical brain regions and the ADI-R, ADOS and ABC scores of subjects with ASD.

#### Resting-state fMRI data preprocessing

The SPM12 package and the Data Processing Assistant for Resting-state fMRI toolbox (DPARSF advanced edition http://rfmri.org/DPARSF) were used for the preprocessing of the resting-state fMRI images. The first 10 images of each subject were excluded to ensure steady-state longitudinal magnetization, then slice time correction was conducted to correct the difference between slices. Head motion correction was applied to correct the head motion related difference between volumes. Subjects with maximum head motion >2.0 mm or 2 degrees were excluded. The corrected images were warped into standard MNI space by DARTEL, smoothed (FWHM = 6 mm) and then detrended, and covariates regression including 5 components of white matter and cerebrospinal fluid signals using CompCor method (Behzadi et al., 2007) and 24 head motion parameter which is defined as 6 original head motion parameter, the first-order difference of the 6 parameters, and the square of these 12 parameters (Friston et al., 1996). Global signal regression was not applied in this study. Then the signals of each voxels were filtered (0.01–0.1 Hz). Prior 1 and latter 2 time points of volumes with framewise displacement (FD) larger than 0.5 mm were discarded (Power et al., 2012). Subjects with remaining volumes <50% of the number of original volumes were discarded, finally 25 ASDs and 27 TDs were included in subsequent analysis. Detailed information of the remaining subjects were list in Table 1.

#### Seed-based functional connectivity analysis

Two clusters with atypical GMV in ASD were detected in the VBM analysis which located in left PCG and left superior temporal gyrus (STG). Then we held a functional connectivity analysis with these two regions as seed regions. The seed ROIs were defined by expanding the center voxel with peak difference in these two clusters to two sphere ROI with 6 mm radius. Functional connectivity (FC) maps for each subject were calculated by extracting the Blood Oxygenation Level Dependent (BOLD) time courses from these two seed regions and the Pearson's correlation coefficients were computed between these two BOLD time courses and the BOLD signals of all other voxels within brain. Then for each subject, a left PCG FC map and a left STG FC map were obtained. The values of FC maps were converted to a normal distribution by Fisher's *Z* transformation.

One sample *t*-test was applied on the left PCG FC maps and left STG FC maps separately to identify the brain regions showing significant connectivity with seed regions (uncorrected *p* < 0.001). This analysis was done in TD group and ASD group separately, for each group we obtained FC masks which indicate the voxels with significant connectivity with each seeds. Then for each seeds, the masks of ASD group and TD group were combined. Two sample *t*-test was utilized on these voxels within the mask obtained in the one sample *t*-test analysis to determine whether the FCs were significantly different between ASD and TD groups using the SPM12 toolbox. Alphasim multiple correction with voxel level *p* < 0.001 and cluster level *p* < 0.05 was applied on the result map of the two sample *t*-test analysis.

The FC values of the regions with significant difference between ASD and TD groups which was defined as the mean FC values of the peak voxel in the atypical clusters with voxels within 6-mm radius were extracted and Pearson correlation analysis was utilized to determine the relationship between the FC values of the atypical brain regions and the ADI-R, ADOS, and ABC scores in ASD group.

## Results

### Structural MRI results

#### Group comparison of gray matter volume (GMV)

**Figure 1:**
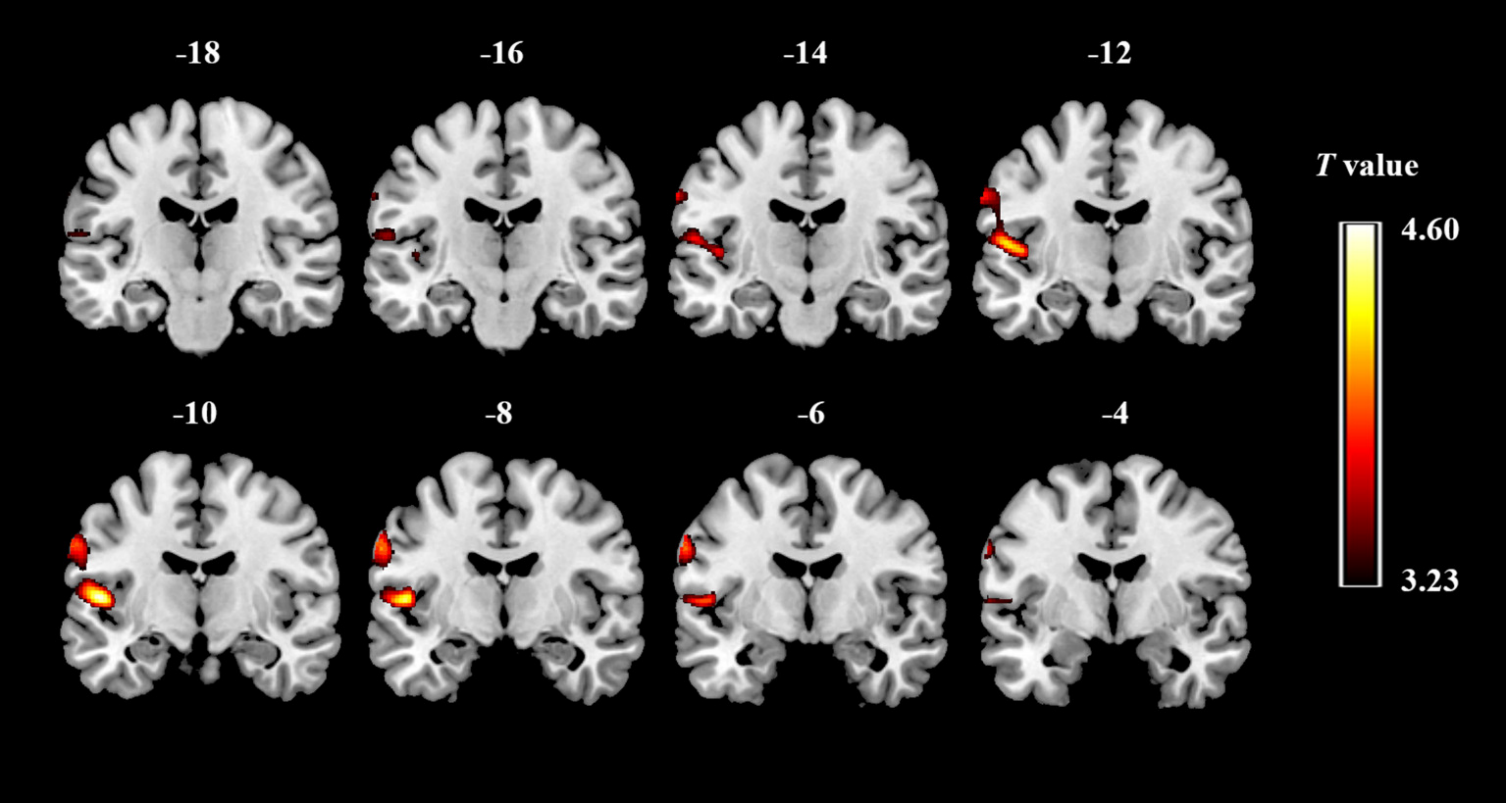
Gray matter regions that discriminate between the groups of ASD and typically developing controls. Red indicates areas of increased gray-matter volume in ASD (ASD > TD).

We observed significant difference in regional GMV between ASD and TD groups. ASD children showed significantly increased GMV in a large cluster (voxel: z = 2,981), including the left STG (x, y, z: −50.5, −9.5, 7.5) and the left PCG (x, y, z: −61.5, −7.5, 31.5) (Figure 1). There was no significantly decreased GMV in brain regions comparing ASD to TD.

#### Behavioral correlations with GMV changes

To examine the relationship between brain structural alterations and core symptoms of ASD children, correlations were calculated between scores on the Autism Diagnostic Observation Schedule (ADOS), Autism Diagnostic Interview-Revised (ADI-R) and ABC and the volumetric data of the brain regions which showed group differences. Analyses revealed that there was no significant correlation between GMV of the left STG and PCG and any of the ADOS, ADI-R and ABC scores, including the total and subscale scores.

### Seed-based resting-state functional connectivity results

#### Functional connectivity with left STG

We did not find any significant between-group differences in functional connectivity between the seed of left STG and whole brain voxels.

#### Functional connectivity with left PCG

##### Within-group functional connectivity map

**Figure 2:**
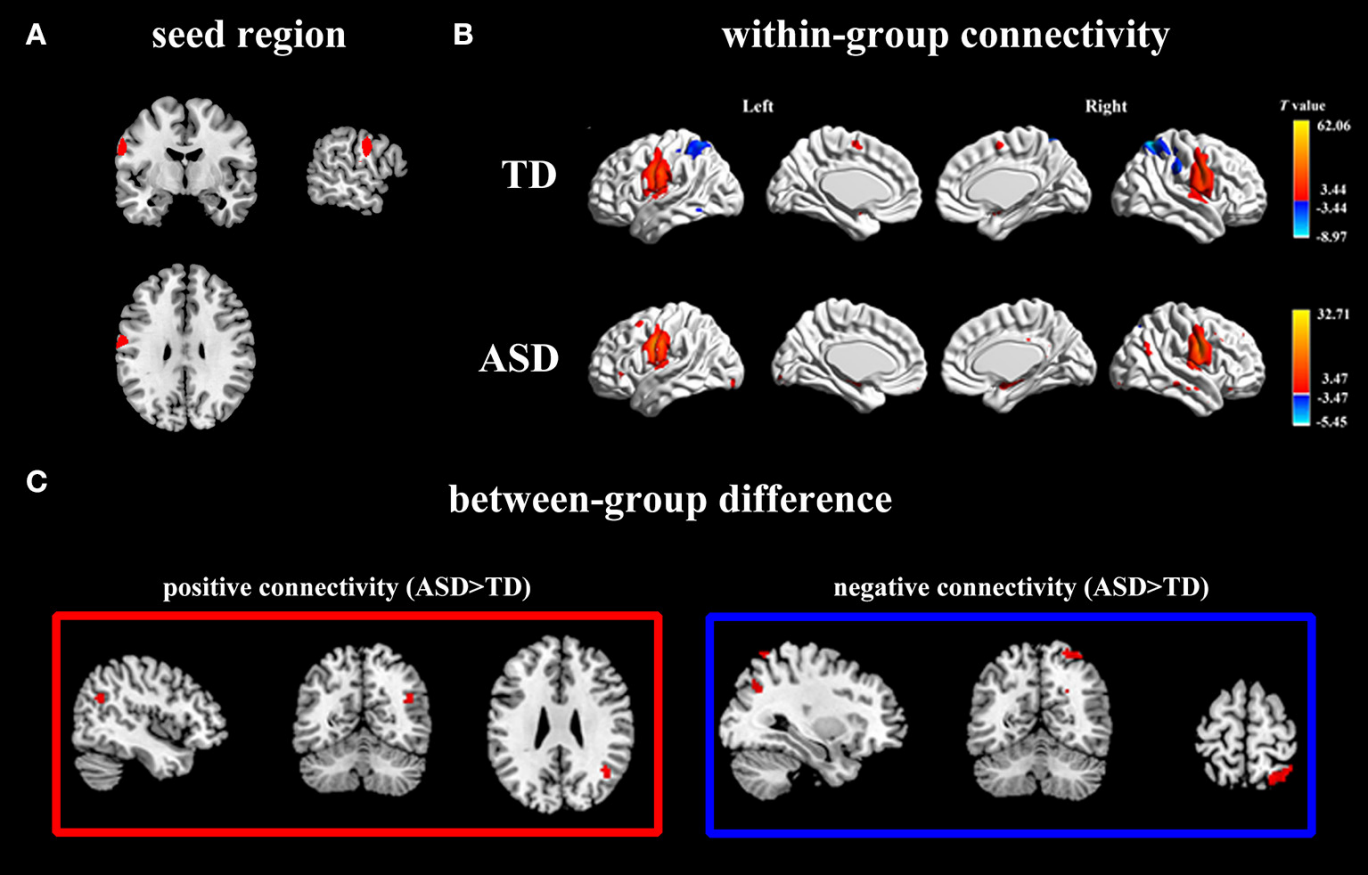
Positive and negative functional connectivity maps with seed of left PCG. **(A)** The left PCG used as seed region. **(B)** Within-group connectivity maps of TD and ASD (*p* < 0.05). Red areas depict voxels with positive correlations, whereas blue areas depict voxels with negative correlations. **(C)** Between-group differences in functional connectivity maps (ASD > TD, *p* < 0.05). The left pictures in the red frame depict voxels of greater positive connectivity with the seed region for the ASD group, while the right pictures in the blue frame depict voxels of greater negative connectivity with the seed region for the ASD group.

In the TD group, activity in left PCG (Figure 2A) was positively correlated with activity in bilateral PCG, superior motor areas, cerebellum as well as right amygdala (Figure 2B; Table 2). For the ASD group, activity in left PCG was positively correlated with a similar network as the TD group except for the bilateral superior motor areas. The ASD group also displayed positive connectivity between the left PCG and the right AG (Figure 2B; Table 2).

**Table 2:** Within-group positive and negative connectivity with seed of left PCG and between-group differences.

	**TD**				**ASD**				**ASD > TD**			
**Region**	**MNI Coordinates**			***t*-value**	**MNI Coordinates**			***t*-value**	**MNI Coordinates**			***t*-value**
	**x**	**y**	**z**		**x**	**y**	**z**		**x**	**y**	**z**	
**POSITIVE CONNECTIVITY**												
Cerebellum_ R	30	−84	−42	5.71	45	−72	−39	6.92				
Cerebellum_ L	−18	−66	−24	8.52	−36	−81	−27	6.61				
Amygdala _ R	27	−9	−12	8.72	21	−6	−12	12.9				
Postcentral_ L	−60	−9	30	62.06	−60	−6	33	32.71				
Postcentral_ R	54	−6	33	24.86	63	−6	30	19.95				
Superior Motor area_ L/R	3	−3	57	5.63	–			NS				
Angular_R	–			NS	42	–57	24	5.22	42	–57	27	4.07
**NEGATIVE CONNECTIVITY**												
Superior Parietal _R	24	−60	66	−8.97	–			NS	24	−60	66	4.27
Superior Parietal _L	−30	−54	63	−6.08	–			NS				
Superior Frontal_Sup_L	–			NS	−18	9	54	−5.45				
Superior Occipital _R	33	−69	39	−3.91	–			NS	33	−69	39	3.98

*x, y, and z refer to the lefteright, anterioreposterior, and inferioresuperior dimensions, respectively; t-value refers to the t score at those coordinates; NS refers to no significant functional connectivity in this area within the group. L, Left; R, right*.

In the TD group, activity in left PCG was negatively correlated with activity in bilateral superior parietal gyrus (SPG) and right superior occipital gyrus (SOG) (Figure 2B; Table 2). For the ASD group, activity in the left PCG was negatively correlated only with left superior frontal lobule (Figure 2B; Table 2).

##### Between-group analyses

Using left PCG as a seed region, we found significantly more positive connectivity with right AG and greater negative connectivity with right SPG and right SOG in ASD group (Figure 2C; Table 2). Relative to the ASD group, the TD group did not show stronger positive or negative connectivity with any regions.

##### Behavioral correlations with functional connectivity in the asd group

To investigate the extent to which altered brain functional connectivities are associated with severity of symptoms in ASD, we further examined the relationship between the specific connectivity strength that exhibited a significant group difference and the ADOS, ADI-R, and ABC scores. Increased functional connectivity between the left PCG and the right AG showed a significant negative correlation with ABC total score and the subscores of the relating, language and self-help and social domains. Meanwhile, increased functional connectivity between the left PCG and the right SPG showed a significant positive correlation with ABC total score and the subscores of the relating, stereotypes and object use, language and self-help and social domains. Consistent with the ABC results, stronger negative connectivity between the left PCG and right SPG in the ASD group also associated with higher ADOS scores on the communication domain as well as the social + communication domain (Table 3). However, none of the behavioral assessment scores was significantly related to the altered functional connectivity strength between the left PCG and the right SOG. We did not find any significant association between altered connectivity strengths and the ADI-R scores.

**Table 3:** Behavioral correlations with abnormal functional connectivity in ASD group (*n* = 24).

	**left PCG-right AG**		**left PCG-right SPG**		**left PCG-right SOG**	
	***r***	***p***	***r***	***p***	***r***	***p***
ABC total score	−0.59	0.00^**^	0.59	0.00^**^	−0.21	0.35
**ABC SUB-SCALE**						
Sensory	−0.32	0.14	0.30	0.18	0.08	0.72
Relating	−0.49	0.02^*^	0.60	0.00^**^	−0.14	0.55
Stereotypes and object use	−0.33	0.14	0.56	0.01^**^	−0.12	0.60
Language	−0.69	0.00^**^	0.47	0.03^*^	−0.34	0.13
Self-help and social	−0.53	0.01^*^	0.46	0.03^*^	−0.24	0.28
**ADOS SUB-SCALE**						
Communication	−0.14	0.54	0.52	0.02^*^	0.25	0.28
Social interaction	−0.20	0.37	0.37	0.10	0.05	0.83
Communication+social interaction	−0.20	0.39	0.47	0.03^*^	0.14	0.55
Stereotyped behaviors and restricted interests	−0.25	0.27	0.20	0.38	−0.19	0.42

p, p-value; r, Pearson correlation co-efficient;

* Indicates statistical significance p < 0.05;

** *indicates statistical significance p < 0.01*.

## Discussion

The present study displayed significant differences of brain structure and functional connectivity between ASD boys aged 3–7 years and matched TDs. First, structural images revealed a cluster exhibiting significant increase in GMV in ASD group, located within the left STG and left PCG. Second, when the left PCG used as seed regions for the functional connectivity analysis, we found significantly higher positive connectivity with right AG and greater negative connectivity with right SPG and SOG in ASD group compared with TDs. Third, the association analysis indicated that connectivity alterations degree is related to symptom severity in ASD children, further demonstrating the robustness of our findings.

### Altered GMV in ASD children

We found a cluster with significantly increased GMV including left STG, one of most commonly reported abnormal brain regions in ASD patients. Prior studies have detected gray matter differences in STG across different life stages in ASD patients, including infants (Xiao et al., 2014), young children (Retico et al., 2016), adolescence (Lim et al., 2015), and young adulthood (Waiter et al., 2004). In agreement with our findings, a recent structural MRI study (Retico et al., 2016) used a relatively large sample size of 2–7 year-old children with ASD also showed a significantly increased GMV in the bilateral STG compared to control subjects. The human STG is critical for speech perception, auditory short-term memory and speech comprehension and has been implicated in social cognition (Leff et al., 2009; Hullett et al., 2016). An fMRI investigation (Eyler et al., 2012) found that the left STG was significantly less responsive to speech stimuli in the ASD group than the typical group suggesting dysfunction of STG during early development in ASD patients. Therefore, abnormalities in the STG are considered relevant to the ASD pathogenesis.

Our results also showed increased GMV in left PCG in ASD children comparing with TDs. The primary somatosensory cortex in the PCG is crucial to exteroceptive and proprioceptive feedback, receiving sensory information via afferent pathways. Although, the imaging literature on the PCG in ASD is currently limited, somatosensory anomalies are commonly observed in each age range of ASD patients and the newly released DSM-5 manual (American Psychiatric Association, 2013) also includes hyporeactivity or hyperreactivity to sensory stimulation as a diagnostic criterion, acknowledging that sensory abnormalities are central in the symptomatology of ASD. It is most likely that somatosensory information processing abnormities may be precipitating factors of behavior problems of ASD patients. Children with ASD are often described as picky or selective eaters and studies revealed that the somatosensory anomalies, such as sensory sensitivity may be a leading factor (Cermak et al., 2010). Besides, previous studies have also demonstrated that children with ASD show an atypical over-reliance on proprioceptive feedback during motor learning (Izawa et al., 2012). Our finding may provide another strong evidence for the pathogenesis of somatosensory anomalies in young children with ASD.

### Altered functional connectivity in ASD children

We compared resting-state functional connectivity between seed areas (left STG and left PCG) with whole-brain voxels for the two groups and revealed pronounced abnormal connectivity between left PCG and right AG, SPG and SOG in ASD children.

#### Altered positive functional connectivity

The ASD group showed significantly higher positive connectivity between left PCG and right AG and this connectivity strength increased with severity of symptoms in ASD children. The AG is a multi-modal hub where converging multisensory information integrated to comprehend and give sense to events, manipulate mental representations and reorient attention to relevant information (Seghier, 2013). Our finding of abnormal connectivity between right AG and left PCG may be the neural substrates leading to the deficit in integrating and coordinating multiple cognitive domains in ASD children. More specifically, research has demonstrated that the right AG plays a key role in tactile processing (Spitoni et al., 2013). Hypo- or hyper-sensitivity to touch and dysfunction of tactile discrimination have been commonly existed in ASD children, seriously affects their normal development and daily life. For example, tactile sensitivity is a leading reason of the stereotyped repetitive interests and behaviors domain in ASD individuals. Significantly increased positive connectivity between left PCG and right AG may cause augmented somatosensory awareness such that proprioceptive and tactile stimuli are actively sought in ASD children.

#### Altered negative functional connectivity

We observed greater negative connectivity between left PCG and right SPG in ASD children. Most of the ABC and ADOS scores showed significant positive associations with the connectivity strength between the left PCG and the right SPG. Therefore, ASD children who showed a greater level of functional connectivity between left PCG and right SPG were more severely impaired in social interaction, communication and self-care domains. The SPG plays a pivotal role in many sensory and cognitive processes, including somatosensory and visuomotor integration, visuospatial attention, mental rotation and the control of socially relevant behaviors (Wang et al., 2015). It is widely acknowledged that ASD patients are dysfunctional during selective attention, mental control, somatosensation mediation and other cognitive operations. Increased negative connectivity between left PCG and right SPG in ASD children may leads to decreased activity in SPG during the integration of sensory processing and other tasks. A recent study (Christakou et al., 2013) found that ASD boys had significantly reduced activation relative to controls in left SPG, which also existed in the patients with attention deficit hyperactivity disorder. Additionally, a structural MRI study (Duerden et al., 2014) reported decreased gray matter thickness in the right SPG and bilateral primary somatosensory cortices in ASD children frequently engaged in self-injurious behaviors in comparison to those with low or no self-injury. A physiology research (Siever et al., 1999) detected reduced metabolism in the right SPG in aggressive patients and individuals with impulsive personality disorders, while a large number of ASD children show similar behaviors with them. Our finding may provide functional evidence for the damaged sensory and cognitive functions in ASD children.

We also found increased negative connectivity between left PCG and right SOG in ASD children. The right SOG, a structure in the dorsal visual stream, is thought to be important in integrating visual processing and other functions (Carlson et al., 2011). A recent rs-fcMRI study (Nebel et al., 2016) in school-age children detected increased negative connectivity between intrinsic activity in visual and motor regions in ASD children reflected reduced integration between visual-motor subnetworks, which is consistent with behavioral evidence that children with ASD struggle with hand-eye coordination and other tasks requiring temporal integration of visual information. Coincidentally, another rs-fcMRI study (Shen et al., 2016) of preschool-age children also indicates that abnormal connectivity between visual cortex and sensorimotor regions was associated with sensory hypersensitivity in the visual/auditory domain in ASD group. There is a generally accepted conclusion that ASD patients showed visual advantage comparing with TDs. Increased negative connectivity between left PCG and right SOG may provide possible neurobiological evidence for the hypothesis that an over-representation of visual information may cause the inhibition of other sensory information processing leading to abnormal functional integration in ASD individuals.

## Limitations

Some limitations should be considered when interpreting the results of the current study. First, the sample size was relatively small and only young male children were included in the cohort. Hence, whether our findings will generalize to adults or female patients with ASD need further verification. Second, we examined volumetric and connectivity changes with neuropsychological scores, but only a few significant correlations were detected. It may due to that the impaired sensory perception and cognition reflected by altered structures and connectivities are not particularly evaluated by the ABC, ADOS, and ADI-R in ASD children. Future studies will have to employ more specialized questionnaires and behavioral assessments to clarify the functional significance of the related brain regions for behavioral problems in ASD.

## Author contributions

JW conceived of the study, performed the data acquisition, and drafted and revised the manuscript. KF were responsible for the acquisition of imaging data, and participated in its design and coordination. LC were involved in the writing and revision of the manuscript, and contributed to the acquisition of clinical data. XD, XG, and HC were responsible for the analysis and interpretation of imaging data and helped to draft the manuscript. QW participated in the acquisition of imaging data. WX was involved in the recruitment and assessment of participants and acquisition of clinical data. HFC and LW conceived of the study, participated in its design and coordination, data acquisition, and helped to revise the manuscript. All authors read and approved the final manuscript.

### Conflict of interest statement

The authors declare that the research was conducted in the absence of any commercial or financial relationships that could be construed as a potential conflict of interest.
